# Nonadherence to Medication Therapy in Haemodialysis Patients: A Systematic Review

**DOI:** 10.1371/journal.pone.0144119

**Published:** 2015-12-04

**Authors:** Saurav Ghimire, Ronald L. Castelino, Nicole M. Lioufas, Gregory M. Peterson, Syed Tabish R. Zaidi

**Affiliations:** 1 Unit for Medication Outcomes Research and Education, Pharmacy, School of Medicine, Faculty of Health, University of Tasmania, Hobart, Australia; 2 Department of Nephrology, Royal Hobart Hospital, Hobart, Australia; Kings College, UNITED KINGDOM

## Abstract

**Background:**

End-stage kidney disease (ESKD) patients are often prescribed multiple medications. Together with a demanding weekly schedule of dialysis sessions, increased number of medicines and associated regimen complexity pre-dispose them at high risk of medication nonadherence. This review summarizes existing literature on nonadherence and identifies factors associated with nonadherence to medication therapy in patients undergoing haemodialysis.

**Methods:**

A comprehensive search of PubMed, Embase, CINAHL, PsycInfo, and Cochrane Database of Systematic Reviews covering the period from 1970 through November 2014 was performed following a predefined inclusion and exclusion criteria. Reference lists from relevant materials were reviewed. Data on study characteristics, measures of nonadherence, prevalence rates and factors associated with nonadherence were collected. The Preferred Reporting Items for Systematic Reviews and Meta-analyses (PRISMA) guidelines was followed in conducting this systematic review.

**Results:**

Of 920 relevant publications, 44 were included. The prevalence of medication nonadherence varied from 12.5% to 98.6%, with widespread heterogeneity in measures and definitions employed. Most common patient-related factors significantly associated with nonadherence were younger age, non-Caucasian ethnicity, illness interfering family life, being a smoker, and living single and being divorced or widowed. Similarly, disease-related factors include longevity of haemodialysis, recurrent hospitalization, depressive symptoms and having concomitant illness like diabetes and hypertension. Medication-related factors such as daily tablet count, total pill burden, number of phosphate binders prescribed and complexity of medication regimen were also associated with poor adherence.

**Conclusions:**

A number of patient-, disease-, and medication-related factors are associated with medication nonadherence in haemodialysis patients. Clinicians should be aware of such factors so that adherence to medications can be optimised in haemodialysis patients. Future research should be directed towards well-designed prospective longitudinal studies developing standard definitions and validating available measurement tools, while focusing on the role of additional factors such as psychosocial and behavioural factors in predicting nonadherence to medications.

## Introduction

End-stage kidney disease (ESKD) is one of the leading causes of mortality with over one million people dying worldwide every year [1]. The incidence of ESKD is increasing globally at an estimated annual rate of 7% [2]. Despite recent advances in the management of ESKD, the cardiovascular and non-cardiovascular mortality risk of chronic haemodialysis patients is 8 times greater than people in the general population [3,4].

The progression of chronic kidney disease to ESKD is often associated with additional comorbidities such as diabetes and cardiovascular diseases [5]. ESKD patients are at high risk of developing imbalances in calcium and phosphate haemostasis, anaemia, hyperlipidaemia, and secondary hyperparathyroidism [6]. Consequently, patients on haemodialysis often require an average of 10–12 regular medications including but not limited to, phosphate binders, vitamin D preparations, calcimimetics, antihypertensives, antidiabetics, erythropoiesis-stimulating agents and iron supplements [7,8]. The resultant complexity of medication regimen in ESKD patients predisposes them to high risk of adverse drug events and subsequent nonadherence [7].

Medication nonadherence can be intentional or unintentional. Intentional nonadherence occurred when patients chose to ignore treatment recommendations by delaying, altering or missing the dosage of prescribed medicines [9]. Unintentional nonadherence, on the other hand, is due to a patient’s lack of understanding, forgetfulness or miscommunication with healthcare providers [10]. Regardless of being intentional or unintentional, medication nonadherence averts patients from gaining the full benefit of the prescribed medications. Furthermore, medication nonadherence in ESKD patients has been associated with increased mortality and hospitalizations [11,12]. Thus, adherence to medication therapy is a key component of the effective management of patients with ESKD [11–14].

To date, there are few review articles addressing specific issues on identifying predictors and determinants of nonadherence to medication therapy in patients undergoing haemodialysis [15–19]. Existing literature is limited to non-systematic reviews examining nonadherence to dialysis treatment as a whole by including medication, dialysis attendance, and diet and fluid restrictions [12,20–22]. It has been observed that about 50% of patients with chronic conditions are nonadherent to medication therapy [23], and the estimates of nonadherence to oral medications in chronic haemodialysis patients ranged from 3 to 80% [18]. A review that specifically focussed on phosphate binder medication in haemodialysis patients reported rates of nonadherence ranging between 22 and 74% [19]. This wide variation in the reported rates of nonadherence was attributed partly due to heterogeneity in definition and methodology of assessing nonadherence in the studies.

The aims of this systematic review were:

1. To identify various methods used to assess nonadherence in patients undergoing haemodialysis
2. To summarize current literature on nonadherence and estimate the prevalence of medication nonadherence in patients undergoing haemodialysis
3. To describe patient-, disease-, and medication-related factors associated with nonadherence in patients undergoing haemodialysis.

## Methods

The Preferred Reporting Items for Systematic Reviews and Meta-analyses (PRISMA) guidelines was followed in conducting this systematic review [24]. The PRISMA checklist is supplied as S1 Appendix.

### Data source and search strategy

We searched PubMed, Embase, CINAHL, PsycInfo, and Cochrane databases covering the period from 1970 through November 2014. Search terms included combinations of Medical Subject Heading (MeSH) terms and keywords like “dialysis/haemodialysis”, “renal replacement therapy”, end-stage renal disease”, “chronic renal failure”, “adherence/nonadherence”, “compliance/non-compliance”, “drug/medication”, and “regimen/schedule.” Details of the initial search strategy are provided in S2 Appendix. A manual search of the references cited in each publication identified from the database search was conducted to identify additional relevant articles.

### Study selection

Titles and abstracts of the articles were screened to include relevant studies. In cases of insufficient information being ascertained from the title or abstract of a paper, a full copy of the article was obtained and screened to determine eligibility. Each article was evaluated for inclusion by two reviewers (SG and RLC) and disagreements between the reviewers were resolved by discussion with the third reviewer (STRZ).

**Fig 1:**
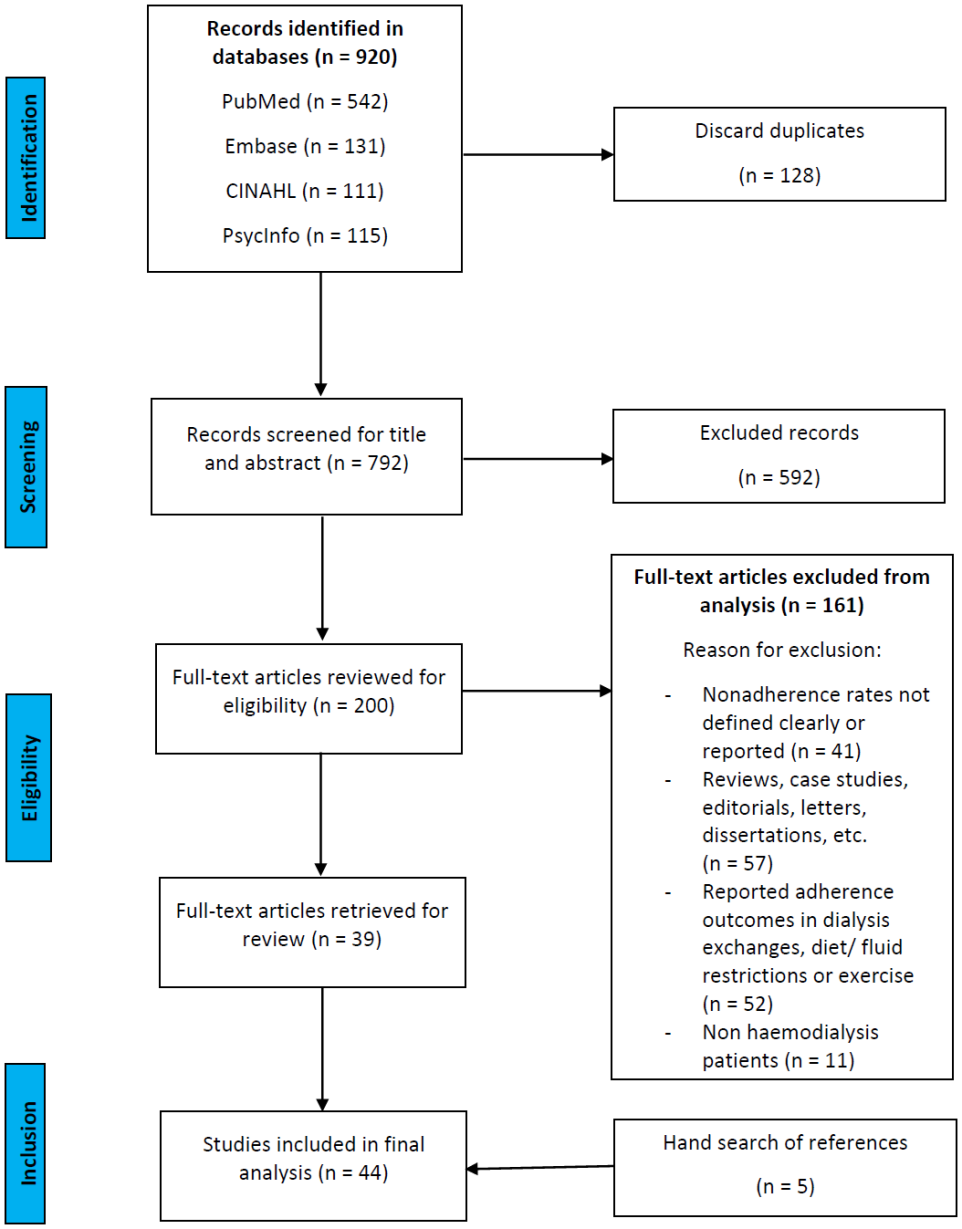
Flowchart of study selection for systematic review.

Studies were included in this review if they fulfilled all of the following criteria: conducted in patient ≥ 18 years, undergoing haemodialysis treatment that included measure(s) of adherence or nonadherence related to medication therapy, and provided numeric results on rates of adherence or nonadherence. All adherence measures like self-report, physician/nurse estimate, pill count, prescription refill, and electronic monitoring were considered if a definition of nonadherence was provided, and nonadherence rates were reported. Studies with the longitudinal or cross-sectional design were included for review. Interventional studies were considered if baseline rates were provided. The publication language was not restricted to English only. Studies were excluded if they reported only adherence outcomes to non-medication interventions such as dialysis exchanges, diet or fluid restrictions, and exercise; did not clearly define or report rates of nonadherence; or if they were reviews, protocols, editorials, letters, or dissertations (Fig 1).

### Data extraction and analysis

Data from the included studies were extracted by one reviewer (SG) followed by verification of all data against the original studies by the second reviewer (RLC). Information extracted included: author, year of publication, country of origin, study design (prospective, retrospective, cross-sectional, etc.), participant characteristics, number of patients, age, gender, types of medications, adherence assessment method, definition of nonadherence, rates of nonadherence, and factors reported to be associated with nonadherence.

Data analysis involved a descriptive summary of included studies. This was mainly attributed due to heterogeneous nature of included studies. Several methods of assessing nonadherence were utilized. We grouped these methods into three broad categories: (1) objective/ direct measures, such as pill count, prescription refill, or using medication event monitoring devices; (2) subjective/ indirect measures that are based on patients’ self-reports or assessment by healthcare professionals and (3) biochemical measures that included measuring of pre-dialysis serum phosphate levels (SPL). To achieve our first objective, we performed frequency counts of each of the methods used to assess nonadherence. For attaining second objective, we grouped reported prevalence of medication nonadherence according to the three overarching subgroup measures and collated findings using a summary bar chart. Our third objective was satisfied by extracting factors associated with nonadherence and presenting them in a tabular format according to statistically significant and non-significant findings across studies per explanatory variables. This method was employed due to inconsistent reporting and heterogeneity of statistical analysis performed in the primary studies.

### Quality assessment

Quality assessment of included studies was independently carried out by two reviewers (SG and RLC) using the Effective Public Health Practice Project (EPHPP) Quality Assessment Tool for Quantitative Studies [25]. The tool addresses six quality domains: selection bias, study design, confounders, blinding, data collection methods, and withdrawals and dropouts. Sections on confounders and blinding were deleted in our adapted version as they were considered irrelevant to this review [10]. Any discrepancies were resolved by discussion with the third reviewer (STRZ).

## Results

### Description of included studies

A flow diagram of the literature search and identification of relevant articles for review is depicted in Fig 1. Overall, 920 potentially relevant articles were identified. In total, 44 articles are summarized and evaluated in this systematic review. Table 1 shows the characteristics of included studies.

**Table 1:** Characteristics of studies included in systematic review.

Study, Year	Region	Patients (% Male)	Mean Age (Years)	Medication	Assessment Method	Nonadherence (NAD) to Medication		Design (Study Quality^a^)
						Definition	Rates, n (%)	
***Nonadherence based on patient self-reports***								
Alkatheri et al., 2014	Saudi Arabia	89 (52.8)	15.0–65.0	PB	Self-report (MMAS-8)	Score < 7 classified as NAD	64 (71.9)	CS (M)
Ossareh et al., 2014	Iran	150 (47.3)	46.5 ± 16.4	PB [CaCO_3_ (n = 136), Al(OH)_3_ (n = 29), SA (n = 26)]	Self-report (SMAQ)	Responding to any of the question with a NAD answer	37 (24.7)	CS (M)
					Self-report (DIPQ)	Taking < 66% of prescribed medication	CaCO_3_, 66 (48.5); Al(OH)_3_, 26 (89.7); SA, 11 (42.3)	
Chater et al., 2014	UK	221 (52.0)	58.1 ± 14.2	PB [CaCO_3_, Al(OH)_3_, SA, Ca(C_2_H_3_O_2_)_2_]	Self-report (7-item MARS)	Score ≤ 28 classified as low adherers	68 (30.8)	CS (M)
Arenas et al., 2013	Spain	181 (56.9)	59.9 (21–86)	PB [CaCO_3_, Al(OH)_3_, Ca(C_2_H_3_O_2_)_2_ SA, LC], CM, Vitamin D	Self-report (SMAQ)	Responding to any of the question with a NAD answer	110 (60.8) (at baseline visit); 79 (71.8) (at 6 month)	P (M)
Santana & Diaz, 2013	Spain	106 (71.0)	61.0 ± 13.0	PB [CaCO_3_, Al(OH)_3_, SA, Ca(C_2_H_3_O_2_)_2_], CM (Cinacalcet)	Self-report (SMAQ)	Responding to any of the question with a NAD answer	40 (37.7)	CS (M)
Theofilou, 2013	Greece	168 (62.5)	62.0	NA	Self-report (5-item MARS)	Score < 20 classified as low adherers	42 (25.0)	CS (M)
Martins et al., 2013	Brazil	502 (66.3)	47.0 ± 13.3	PB	Interview	Reporting missed dose	330 (65.7)	CS (M)
Garcia-Llana et al., 2013	Spain	30 (60.0)^b^	60.6 ± 16.7	AHT (n = 17); PB (n = 25)	Self-report (MGLT-4)	Responding to any of the question with a NAD answer	AHT, 15 (90.9); PB, 17 (68.4)	CS (M)
Rosenthal Asher et al., 2012	USA	85 (40.0)^b^	55.9 ± 13.2	NA	Self-report (ITAS-M)	Score ≤ 9 classified as low adherers	11 (13.0)	P (M)
Wileman et al., 2011	UK	76 (60.5)	63.1 ± 15.4	PB	Self-report (MAQ)	Responding to any of the question with a NAD answer	11 (14.5)	CS (M)
Neri et al., 2011	Italy	1,238 (-)	61.7 ± 14.5	NA	Self-report (MGLT-4)	Responding to any of the question with a NAD answer	644 (52.0)	P (M)
Cukor et al., 2009	USA	65 (46.0)^b^	51.1 ± 13.0	NA	Self-report (ITAS-M)	Score ≤ 9 classified as low adherers	24 (37.0)	P (M)
Garcia et al., 2008	Spain	47 (63.0)^b^	70.0 ± 14.5	PB	Self-report (MGLT-4)	Responding to any of the question with a NAD answer	24 (52.3)	CS (M)
Hirth et al., 2008	Multinational^c^	7,852 (-)	62.4 ± 14.6	AHT, PB, CM	Self-report	Reporting cost related NAD	1052 (13.4)	CS (M)
Lindberg et al., 2007	Sweden	150 (60.0)^b^	63.6 ± 14.3	AHT, PB, CM, HDS	Self-report	Differences in the self-reported drug and prescription record	120 (80.4)	CS (W)
Holley & DeVore, 2006	USA	39 (44.0)^b^	67% over 50	NA	Self-report	Missing prescription filling	11 (22.0)	CS (W)
						Reporting missed dose	21 (39.0)	
Rahman & Griffin, 2004	USA	270 (53.0)	60.4 ± 16.0	AHT (n = 205)	Self-report	Reporting missed dose	47 (23.0)	CS (M)
Horne et al., 2001	UK	47 (48.9)	49.0 ± 17.3	NA	Self-report (BMQ)	Responding to any of the question with a NAD answer	27 (57.4)	CS (M)
Caraballo Nazario et al., 2001	USA	53 (41.7)	51.5 ± 14.3	AHT, PB	Structured Interview	Reporting missed dose	39 (75.0)	CS (M)
Gago et al., 2000	Spain	121 (56.2)	62.8 ± 12.6	AHT (n = 49); PB [CaCO_3_ (n = 104); Al(OH)_3_ (n = 39)]	Self-report	Differences in the self-reported drug and prescription record	AHT, 6 (12.5); CaCO_3_, 14 (14.0); Al(OH)_3_, 4 (12.5)	CS (W)
Kaplan et al., 1994	USA	30 (40.0)	40.5 (14–69)	AHT, PB	Self-report	Reporting missed dose	20 (66.7)	CS (M)
Blanchard et al., 1990	USA	40 (50.0)	50.4 ± 16.4	Ca Supplements, PB, Vitamins	Self-report	Reporting missed dose	11 (27.5)	P (M)
***Nonadherence based on objective measures***								
Park et al., 2014	USA	11,732 (56.2)	69.4 ± 12.7	AHG (n = 3,819); AHT (n = 9,863); AL (n = 4,607); CM (n = 2,436); PB (n = 7,753)	MPR	MPR < 80% (Poor adherence)	AHG: 2,338 (61.2); AHT: 4,098 (41.5); AL: 2,118 (46.0); CM: 1,587 (65.1); PB: 6,068 (78.3)	R (M)
Porter 2013	USA	96 (53.1)	52.5 ± 14.6	PB (SA), CM, Vitamin D	Refill per EMR	Medication course either not started or partially completed	35 (36.5)	R (M)
Lee at al., 2011	USA	4,923 (53.3)	61.8 ± 13.8	CM (Cinacalcet)	Refill	≥ 180 days refill gap	2,247 (45.6)	R (M)
					MPR	MPR < 0.8 (Poor adherence)	1,304 (26.5)	
Gincherman et al., 2010	USA	79 (43.0)	51.0 ± 13.0	CM (Cinacalcet)	MPR	MPR < 0.8 (Poor adherence)	56 (70.9)	R (M)
Chiu et al., 2009	USA	233 (58.0)	52.9 ± 14.7	PB [CaCO_3_, Al(OH)_3_, Ca(C_2_H_3_O_2_)_2_, SA, LC]	Pill count	Taking < 80% of prescribed pills	144 (62.0)	CS (M)
Curtin et al., 1997	USA	135 (47.0)	63.2 ± 13.8	AHT (n = 83); PB (n = 98)	MEMS	Instance of bottle opening	AHT, 77 (92.8); PB, 96 (97.9)	P (M)
***Nonadherence based on biochemical measures***								
Wileman et al., 2015	UK	112 (61.6)	60.5 ± 16.9	PB	SPL	SPL > 5.0 mg/dL	79 (70.5)	CS (M)
O’Connor et al., 2008	UK	73 (60.3)	51.9 ± 14.7	PB	SPL	SPL ≥ 5.5 mg/dL	40 (55.0)	P (M)
Tijerina, 2006	USA	26 (0.0)	30–56	PB	SPL	SPL > 6.0 mg/dL	16 (61.5)	CS (M)
Saounatsou, 1999	Greece	60 (53.3)	49.4	PB	SPL	SPL > 5.0 mg/dL	17 (28.3)	CS (M)
Leggat et al., 1998	USA	6,251 (49.7)	57.8 ± 15.5	PB	SPL	SPL > 7.5 mg/dL	1,383 (22.1)	R (M)
Bame et al., 1993	USA	1229 (47.1)	56.7 (18–90)	PB	SPL	SPL > 6.0 mg/dL	612 (49.8)	CS (M)
Weed-Collins & Hogan, 1989	USA	30 (43.0)	25–80	PB	SPL	SPL > 5.5 mg/dL	19 (64.0)	CS (M)
Betts & Crotty, 1988	USA	46 (33.0)	41–60	PB	SPL	SPL > 5.0 mg/dL	35 (76.1)	CS (M)
Cummings et al., 1982	USA	116 (54.0)	54.8 (21–76)	PB	SPL	SPL > 5.5 mg/dL	81 (70.0)	CS (M)
Wenerowicz et al., 1978	USA	19 (68.4)	36.0 (19–70)	PB	SPL	SPL > 4.5 mg/dL	13 (68.4)	CS (M)
***Nonadherence based on multiple measures***								
Sgnaolin et al., 2012	Brazil	65 (49.2)	59.1 ± 14.7	AHT, PB	SPL	SPL > 5.5 mg/dL	25 (38.5)	CS (M)
					Self-report (MGLT-4)	Responding to any of the question with a NAD answer	36 (55.4)	
Chan et al., 2012	Malaysia	188 (48.9)	58.2 ± 10.5	PB	SPL	SPL > 5.0 mg/dL	63 (33.5)	CS (M)
					Self-report (DDFQ)	Score ≤ 3 classified as low adherers	93 (49.5)	
Arenas et al., 2010	Spain	165 (63.0)	65.2 ± 14.7	PB [Al(OH)_3_, Ca(C_2_H_3_O_2_)_2_, SA]	SPL	SPL > 5.5 mg/dL	23 (13.9)	CS (M)
					Self-report (SMAQ)	Responding to any of the question with a NAD answer	66 (40.0)	
Lin & Liang, 1997	China	86 (-)	55.0 (45.0)	PB	MCA	SPL > 5.0 mg/dL	52 (61.0)	CS (M)
						Nurse assessment	26 (30.8)	
						Self-report	20 (23.6)	
Cleary et al., 1995	USA	51 (45.1)^b^	51.0 ± 17.0	AHT, PB, Vitamin D	SPL	SPL > 4.5 mg/dL	23 (45.1)	CS (M)
					Structured Interview	Reporting missed dose	30 (60.0)	
Curtin et al., 1999	USA	135 (46.7)	63.2 ± 13.8	AHT (n = 69), PB (74)	Self-report (BMQ)	Overdosing, under dosing, or missing an entire day’s dose	AHT, 14 (20.3); PB, 34 (45.9)	CS (M)
					Pill count	Number of pills added at each refill	AHT, 63 (91.3); PB, 73 (98.6)	
					MEMS	Instance of bottle opening	AHT, 66 (95.7); PB, 72 (97.3)	

Note: Conversion factor for unit: SPL in mg/dL to mmol/L, x0.3229. Abbreviations: AHG, antihyperglycemics; AHT, antihypertensives; AL, antilipidemics; BMQ, brief medication questionnaire; CM, calcimimetics; DDFQ, dialysis diet and fluid nonadherence questionnaire; DIPQ, drug intake percentage questionnaire; EMR, electronic medical record; HDS, herbal and dietary supplement; ISAI, Iowa self-assessment inventory; ITAS-M, modified immunosuppressive therapy adherence scale; LC, lanthanum carbonate; MAQ, medication adherence questionnaire; MARS, medication adherence report scale; MCA, multi-method compliance assessment (including: laboratory assessment, nurse assessment, and patient self-report); MEMS, medication event monitoring system; MGLT-4, Morisky 4-item Green Levine test; MMAS-8, Morisky 8-item medication adherence scale; MPR, medication possession ratio; PB, phosphate binder; SA, sevelamer hydrochloride; SMAQ, simplified medication adherence questionnaire; SPL, pre-dialysis serum phosphate level; Study design (CS, cross-sectional; P, prospective; R, retrospective); NA, not available.

^a^Effective public health practice project (EPHPP) quality assessment tool for quantitative studies. Study quality (S, strong; M, moderate; W, weak).

^b^Subsample of haemodialysis patients.

^c^Twelve industrialized countries (Australia/ New Zealand, Belgium, Canada, France, Germany, Italy, Spain, Sweden, and UK, twenty facilities each; Japan, sixty facilities; and USA, eighty facilities).

Half (n = 22) of the studies [14,26–46] were conducted in North America, 15 were carried out in Europe [23,47–60], four were conducted in Asia [61–64], and two studies were performed in South America [65,66]. One included study had a multicentre site in 10 different countries [67].

Most of the included studies (n = 32) were cross-sectional in design [32–36,38–41,43–51,53,54,56–67], with another seven of prospective nature [23,28,31,37,42,52,55], and five having retrospective study design [14,26,27,29,30].

The sample size greatly varied from a minimum of 19 participants [46] to a maximum of 11,732 participants [26]. Overall, half (n = 22) of the included studies had a sample size of more than 100 participants [14,23,26,29,32,35,37,38,41,45,47–50,52,54,57,58,61,63,66,67]. Moreover, five studies had more than 1000 participants each [14,26,29,41,67]. All included studies comprises of ESKD patients receiving treatment at hospital-based outpatient haemodialysis centres.

### Assessment of nonadherence

Half of the studies (n = 22) exclusively applied subjective measures based on patients’ self-report to assess nonadherence. However, the specific method of subjective assessment differed across studies. Thirteen studies [23,28,31,48–53,56,59–62] used self-reported measures with a validated questionnaire (Brief Medication Questionnaire (BMQ); Drug Intake Percentage Questionnaire (DIPQ); Modified Immunosuppressive Therapy Adherence Scale (ITAS-M); Medication Adherence Report Scale (MARS); Morisky 4-item Green Levine Test (MGLT-4); Morisky 8-item Medication Adherence Scale (MMAS-8); Medication Adherence Questionnaire (MAQ); and Simplified Medication Adherence Questionnaire (SMAQ)) whereas, 9 studies utilised self-report by patient interview or non-validated questionnaires [34–36,40,42,57,58,66,67].

Studies solely utilising biochemical measures of assessing nonadherence, based on pre-dialysis SPL, accounted for less than 25.0% (n = 10) of our included sample [14,33,41,43–47,55,59]. Furthermore, the least utilized method of assessing nonadherence to medication in haemodialysis patients was directly (13.6%, n = 6), that included either pill count or using electronic monitoring devices [26,27,29,30,32,37].

Five out of the six studies that used two or more instruments to measure nonadherence employed subjective (patient self-report) and biochemical measures (pre-dialysis SPL) [39,54,63–65]. The remaining one study [38] integrated subjective with objective measures like pill count and electronic monitoring system, respectively.

### Definitions of nonadherence

Studies reported wide variation in the definitions for each (subjective, objective, and biochemical) measure of nonadherence.

Subjective measures that used validated questionnaires defined nonadherence based on adherence rating scales [28,31,45,47,48,50,62,63]. On the contrary, studies relying on non-validated questionnaires or interviews defined nonadherence by self-reported missed doses [34–36,39,40,42,66], cost-related nonadherence [67], or discrepancies in the self-reported adherence and prescription records [57,58].

For objective measures, the nonadherence definition were based on pill count (taking less than 80% [32] of prescribed medication), prescription refill frequency [27], instances of bottle opening as detected by using medication event monitoring devices [37,38], and medication possession ratio (MPR), defined as the number of doses dispensed in relation to the dispensing period with a cut-off value of 80% [26,29,30].

Studies considering biochemical measures for estimating nonadherence showed variation in their definition. The upper limit of the acceptable range for pre-dialysis SPL were reported from 4.5 mg/dL [39,46] to 7.5 mg/dL [14]. Though, most of the studies (66.7%, n = 10) considered pre-dialysis SPL acceptable at the upper limit of 5 mg/dL [44,47,52,59,63,64] to 5.5 mg/dL [43,45,53–55,65]. A clinical proxy measure like SPL is often influenced by clinical variables and dietary intake, and, therefore, could confound an exploration of the relationship between serum phosphate and adherence outcomes [47]. During our analysis we found five studies that employed both pre-dialysis SPL and patient self-report measures to assess the adherence outcomes [39,54,63–65] (Table 1).

### Prevalence of nonadherence to medication

**Fig 2:**
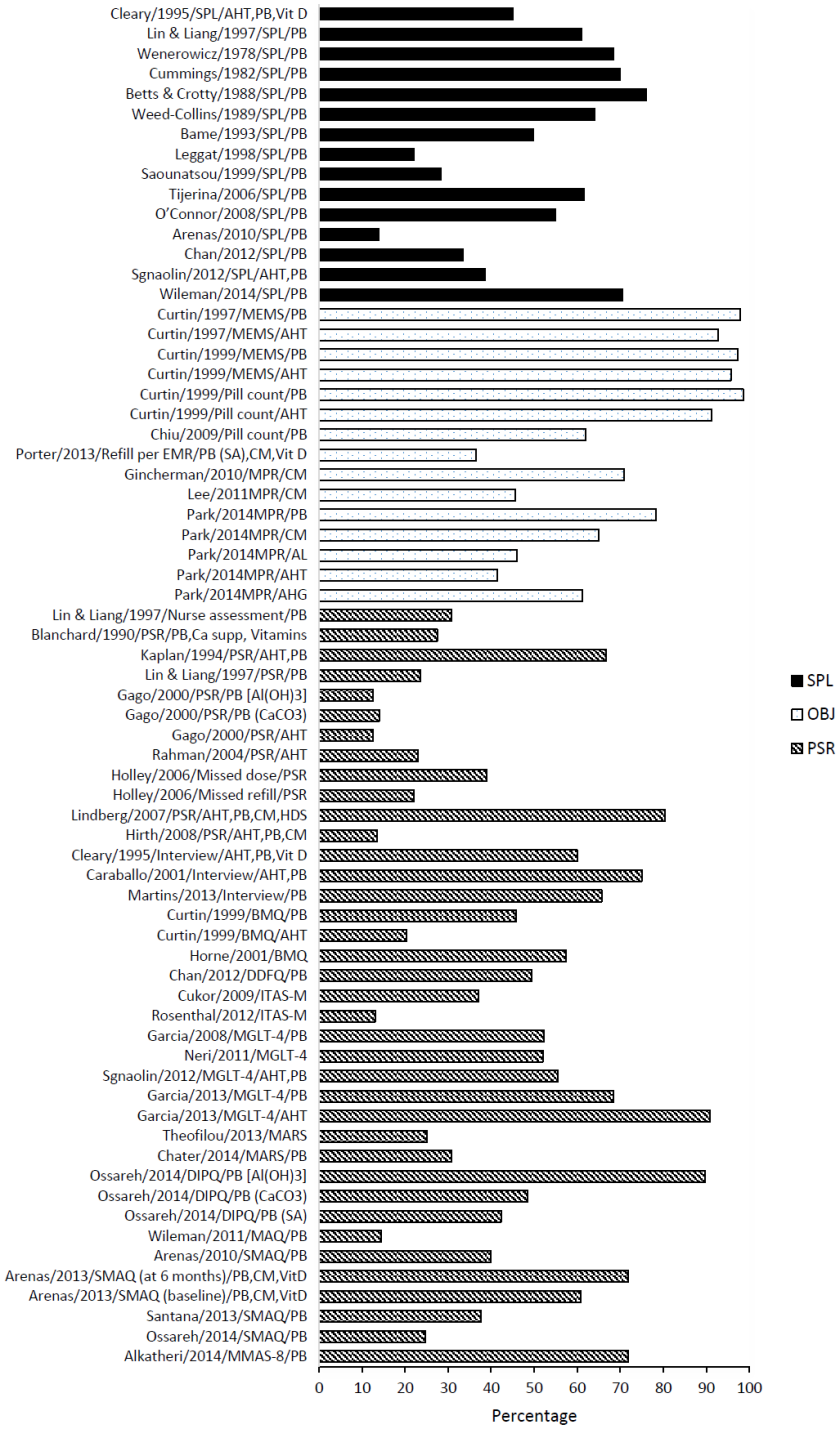
Prevalence rates of medication nonadherence in HD patients. Abbreviations: AHG, antihyperglycemics; AHT, antihypertensives; AL, antilipidemics; BMQ, brief medication questionnaire; CM, calcimimetics; DDFQ, dialysis diet and fluid nonadherence questionnaire; DIPQ, drug intake percentage questionnaire; EMR, electronic medical record; HD, haemodialysis; HDS, herbal and dietary supplements; ITAS-M, modified immunosuppressive therapy adherence scale; MARS, medication adherence report scale; MGLT-4, Morisky 4-item Green Levine test; MMAS-8, Morisky 8-item medication adherence scale; MPR, medication possession ratio; MAQ, medication adherence questionnaire; MEMS, medication event monitoring system; OBJ, objective measure of adherence; PB, phosphate binders; PSR, patient self-reported adherence; SA, sevelamer hydrochloride; SMAQ, simplified medication adherence questionnaire; SPL, pre-dialysis serum phosphate levels.

In general, rates of nonadherence to medication in haemodialysis patients ranged from 12.5% to 98.6%. This variation was primarily observed due to different measures and definitions employed in estimating nonadherence rates. Fig 2 shows the prevalence rates of medication nonadherence in haemodialysis patients according to the three subgroup measures of adherence (subjective, objective, and biochemical) and also consolidates prevalence rates for similar measures within the three overarching subgroups.

The most frequently studied renal-specific medications were phosphate binders (76.1%, n = 35), with eight studies [27,32,48,49,52,54,58,61] specifically mentioning the types of phosphate binders prescribed (aluminium hydroxide, calcium acetate, calcium carbonate, lanthanum carbonate, and sevelamer hydrochloride). Other medications studied included, antihypertensives (27.3%, n = 12), calcimimetics (17.4%, n = 8). Fewer studies (9.1%, n = 4) estimated nonadherence to antidiabetic agents, antidyslipidaemic drugs, and calcium and vitamin D supplement products. Six studies did not specifically mention the types of medications studied [23,28,31,34,50,60].

Nonadherence to phosphate binders ranged from 13.9–98.6%, with an average of 52.5%. The mean percentage of patients classified as non-adherent assessed by pre-dialysis SPL, subjective measures and objective measures were 28.6%, 47.9% and 78.4%, respectively.

The estimates of nonadherence to antihypertensive medication in haemodialysis patients ranged between 12.5% and 95.7% (mean 38.2%). When assessed using different measures of nonadherence like patient self-report and objective measures, the mean prevalence rates were 24.3% and 38.5%, respectively. The rate of nonadherence to other medications such as antidiabetics and antidyslipidaemics were 61.2% and 46.0%, respectively.

Among five studies [39,54,63–65] that used composite methods for measuring adherence, the rates of nonadherence varied greatly depending on the types of adherence measure used. The rates of nonadherence were lower when assessed using pre-dialysis SPL (ranged from 13.9% to 45.1%), whereas the same studies reported higher rates of nonadherence when using patient self-report measures (ranged from 40.0% to 60.0%) [39,54,63,65]. The opposite was true with one study where the rate of nonadherence was higher with pre-dialysis SPL (61.0%) and lower with patient self-report measures (23.6%) [64] (Table 1).

### Factors associated with nonadherence

A total of 38 studies reported factors associated with nonadherence in patients undergoing haemodialysis. Data synthesis on the factors associated with nonadherence were based on the statistical significance and the direction (positive or negative) of the association. The majority of studies relied on a univariate analysis to explore the factors associated with nonadherence with only 15 studies using multivariate analyses [14,23,26,28,29,31,32,41,47,48,53–55,61,63]. A quantitative summary of statistically significant factors and their logical categorisation is presented as Table 2.

**Table 2:** Factors associated with nonadherence (N = 38).

Factors	No of studies	Significant association with measures of nonadherence^a^			References
		SPL	PSR	PC/ MEMS	
***Socio-demographic variables***					
Age	27				
*Younger*		8	8		[23,27,41,44,45,47,48,53,54,57,61–63,65]
*Older*		1	2	1	[14,31,32,50]
Gender	22				
*Male*			1		[61]
*Female*			2		[23,53]
Low education (high school)	15		1		[62]
Ethnicity (non-Caucasian)	7	1	1	2	[14,37,38,48]
Marital status (single, divorced or widowed)	6		2		[50,62]
Employment status (unemployed)	6		1		[50]
Support from health care provider	2		2		[23,45]
Family problems (illness interfering with family life)	2		1		[45]
Smoker	1	1			[14]
***Clinical variables***					
Long-term on HD	16		3		[23,50,63]
Comorbidity (DM, HTN)	9	1	1		[54]
Number of hospitalization	2		1		[23]
***Psycho-social variables***					
Depressive symptoms	6		4		[28,31,50,61]
Belief about medicine	5				
*Concern*		1	2		[48,53,60]
*Benefit*		1	1		[45]
*Necessity*		1	3		[47,48,53]
*Necessity-concern differential score*			2		[47,53]
Health locus of control^b^	3	2	1		[46,64]
*Internal*			1		[50]
*Doctors*			1		[50]
Emotional representation	1	1			[55]
***Medication related factors***					
Knowledge about medicine	5	1	1		[45,63]
Number of prescribed medicines	3			1	[37]
Daily tablet count	2	1	1		[23,54]
Total no of PB prescribed	2	1	1		[54]
Total pill burden	2		1	1	[23,32]
Pill burden from PB	1			1	[32]
PB equivalent dosage	1	1			[47]
Regimen complexity (frequency and dosage schedule)	1	1			[45]
Drug coverage by insurance	1			1	[26]
Health care cost (inpatient)	1			1	[29]

Abbreviations: DM, diabetes mellitus; HD, haemodialysis; HTN, hypertension; MEMS, medication event monitoring system; PB, phosphate binders; PC, pill count; PSR, patient self-report; SPL, pre-dialysis serum phosphate level.

^a^Level of significance (p < 0.05, p < 0.01, and p < 0.001) varies between studies.

^b^Defined as having high expectation that one’s actions will have a causal relationship with the consequences produced.

Taking into account the relative number of studies that explored variables associated with nonadherence and the actual studies that found a significant association, we have identified a number of variables that are likely to influence medication adherence in haemodialysis patients. A number of demographic factors were found to be significantly associated with nonadherence. Age was one of the most frequently reported variable. Although younger age was commonly associated with nonadherence, four studies found nonadherence prevalent in older population as well. Other factors significantly associated with measures of nonadherence were: non-Caucasian ethnicity; illness interfering family life; being a smoker; and living single and being divorced or widowed. Very few studies found female gender, low education, and unemployment to be significantly associated with nonadherence. Support from healthcare providers had a significant positive effect on adherence to medication therapy.

Longevity of haemodialysis (5 or more years on dialysis) was reported as the most common clinical factor, but only three studies [23,55,63] found it to be significantly associated with nonadherence. Other clinical variables influencing adherence were having a concomitant illness like diabetes and hypertension, and recurrent hospitalization (Table 2).

The psycho-social variables that were identified to influence nonadherence included: depressive symptoms; negative belief about medicines (concern, benefit, necessity, and necessity-concern differential score; calculated by subtracting the concerns subscale scores from the necessity subscale score, where the negative scores indicate that patients rate their concerns about medication above their beliefs in the necessity of taking it) [45,47,48,53,60]; health locus of control, defined as having high expectation that one’s actions will have a causal relationship with the consequences produced [46,50,64]; and emotional representation i.e. emotional distress specific to the illness (Table 2).

Overall, nine studies [23,26,29,32,37,45,47,54,63] reported medication-related factors that were found to be significantly associated with nonadherence. These included daily tablet count, knowledge about medicines, total pill burden, total number of phosphate binders prescribed, phosphate binder equivalent dosage (the relative phosphate binding coefficient based on weight of each binder that can be estimated relative to calcium carbonate), pill burden from phosphate binder, medication regimen complexity (frequency and dosage schedule), drug coverage by insurance, and health care cost as inpatients (Table 2).

Fewer studies [45,47,54], evaluated factors associated with nonadherence using more than one measure of nonadherence (pre-dialysis SPL and patient self-report). The factors that showed significant correlation with both patient self-reported adherence and pre-dialysis SPL were: age [47,54]; comorbidity [54]; total number of phosphate binders prescribed [54]; belief about phosphate binder medicine (necessity) [47]; and belief about medicine (benefits) [45]. However, belief about phosphate binder medicine (concern) was not significantly associated with both measures of adherence [47], suggesting that although patients had some concerns about their phosphate binder medicines this did not appear to consistently influence their medication-taking behaviour.

### Perceived barriers of adherence to medication

Eight studies reported patients’ perceived barriers to adherence with medication therapy. The most common reasons given by the patients to explain nonadherence were: forgetfulness (n = 6 studies), poor tolerance or side effects (n = 4 studies), pill burden (n = 3 studies), and large tablet size (n = 2 studies). Other reasons included: unpalatable taste; medication regimen complexity (frequency and dosage schedule); difficulty in opening the medication container; prescription refilling; medication cost; transportation; knowledge about phosphate binder medicines; diet and fluid restrictions; knowledge about importance of taking medicines; lack of interest; monotony; being away from home; and social discomfort [34,35,39,40,43,49,52,63].

### Study quality

Based on the EPHPP Quality Assessment Tool, most studies (n = 41) were rated as moderate quality (Table 1). The reasons behind this moderate rating were, weak study design largely based on cross-sectional data [32–36,38–41,43–51,53,54,56–67], using non-validated measures of data collection like patient interviews or lacking reliability data from the use of validated measures [27,28,31,34–36,38–40,42,48–52,56–58,61,62,65–67], and failure to report withdrawals and dropout rates of participants completing the study [14,23,26,27,29–31,33,34,40,41,43,45,46,48,54–59,61,64,67].

## Discussion

The present systematic review summarized findings from 44 studies over a period of three decades to identify factors associated with nonadherence to medications in patients undergoing haemodialysis. Given the absence of a unified standardised approach to measuring nonadherence [68], the current review observed significant variability in the methodological quality of included studies.

A number of methods of assessing nonadherence to medication were observed in this review, such as objective measures of pill count, subjective measures of patient self-reports and biochemical measures of measuring pre-dialysis SPL. Half of the studies exclusively applied subjective measures based on patients’ self-report to assess nonadherence compared to the two previous reviews [18,19] that reported measurement of SPL as the most frequent method. This transformation may be due to the availability of validated medication adherence scales to measure nonadherence in clinical practice [69]. Additionally, limitations of SPL are increasingly being recognised as it can be influenced by non-medications related factors, such as adherence to dietary restrictions, dialysis attendance, residual renal function, hormonal and acid-base balance, and type and intensity of dialysis treatment [19,70].

Discrepancies in defining nonadherence were observed among studies that used subjective measures with non-validated questionnaires [34–36,39,40,42,66], and biochemical measures like pre-dialysis SPL [14,39,46]. Owing to these inconsistent definitions, wide variation in the reported rates of nonadherence was observed. A study defining the acceptable range of pre-dialysis SPL at a higher cut-off value of 7.5 mg/dL reported the lowest rates of nonadherence (22.1%) [14], whereas the study adopting a lower cut-off value of 4.5 mg/dL reported one of the highest rates of nonadherence (68.4%) [46]. Combining information across studies becomes problematic when a patient defined as adherent based on certain criteria in one study would be defined as non-adherent based on different criteria in another study [71]. Hence, there is a need for the consensus on defining or assessing medication adherence to study the problem effectively, understand the underlying factors, and develop and test interventions to improve adherence.

Overall, the prevalence rates of nonadherence to medication ranged between 12.5% and 98.6%, which is comparatively higher than with other chronic conditions like diabetes (prevalence rates ranged from 6.9% to 61.5%) [72], schizophrenia-spectrum disorders (5.0% to 52.8%) [73], and chronic skin conditions, like psoriasis (33.4% and 78.4%) [74]. Nonadherence rates in haemodialysis patients are higher in comparison to other dialysis modalities such as peritoneal dialysis (PD) that ranged from 3.9% to 43.0% [10]. These divergent findings between two modalities of dialysis treatment might have been influenced by the intermittent nature of maintenance haemodialysis sessions that requires more stringent dietary and medication requirements as compared to PD. Other factors include that PD is often a starter therapy, and patients may not been sick for as long as those on haemodialysis [75]. Also some PD patients are transplanted or eventually switch to haemodialysis. This selects out often a younger population who may have a lesser dialysis vintage, disparity in health literacy and dialysis knowledge [3].

A number of demographic and clinical factors were found to be significantly associated with nonadherence. Not surprisingly, the findings correspond with the results of a systematic review on determinants of patient adherence conducted by Kardas et al [76]. Besides that, different aspects of beliefs about medicines were found to be possible barriers for adherence that includes necessity, concern, and benefits from the medication therapy. Patients who expressed lower necessity beliefs and greater concerns about potential adverse effects of medication were more likely to be nonadherent [45,47,48,53,60]. A significant majority of haemodialysis patients are prescribed with phosphate binders and antihypertensive medications that account for high pill burden [32], are associated with adverse effects, and results into nonadherence [32]. Phosphate binders often cause constipation and gastrointestinal discomfort to the patients [77]. Similarly, antihypertensive medicines potentially add to hypotension post-dialysis treatment [78], and patients can cease these medications due to the haemodynamic effects they experience. Therefore, taking account of patients’ necessity beliefs and concerns in prescribing and treatment review is essential to support informed choice and optimal adherence to prescribed treatment [79].

The need for lifelong complex medication regimens can contribute to nonadherence [6]. Surprisingly, among the nine studies that assessed medication-related factors, only one study identified that medication regimen complexity (frequency and dosage schedule) was significantly associated with nonadherence [45]. Medication regimen complexity can be measured with the medication regimen complexity index (MRCI), a validated instrument developed by George et al [80]. Unfortunately, in most chronic illness, including ESKD, researchers have not measured regimen complexity until recently [81]. Change in MRCI scores following an intervention has been studied in diabetes, elderly and home haemodialysis patients [82–84]. Initiatives aiming to improve medication adherence should consider the above-mentioned determinants to ensure patients are actively involved in designing medication regimen considering the relative contribution of each medicine to the regimen complexity.

This study has some limitations. They are mostly related to the source publications included in this review. The majority of the reviewed studies were cross-sectional in design, considered to be of limited suitability for assessing adherence behaviour [73]. Furthermore, the reverse causation bias [85] cannot be ruled out in cross-sectional studies, therefore, readers are encouraged to exercise caution in the interpretation of the findings from this review. An examination of clinical outcomes and consequences of nonadherence to medication therapy was beyond the scope of this review. Due to the time and resource limitations, we predominantly relied on the full-text articles published in peer-reviewed journals and did not search conference proceedings for relevant abstracts. Nevertheless, the studies included in this review represent a diverse community of patients from wide geographic locations. Furthermore, more than half of the included studies had large sample sizes above 100 participants.

## Conclusion

Nonadherence to medication therapy is a significant issue in patients undergoing haemodialysis. Differences in definitions and tools to measure nonadherence are widespread in the current literature. This necessitates a consensus on defining or assessing medication nonadherence in order to study underlying issues effectively, understand barriers to adherence properly, and develop and test intervention measures to improve adherence in haemodialysis patients. Abiding by the definition of clinical targets for biochemical measures like pre-dialysis SPL as recommended by international clinical practice guidelines such as Kidney Disease Improving Global Outcomes (KDIGO), National Kidney Foundation- Kidney Disease Outcomes Quality Initiative (NKF- KDOQI), or Kidney Health Australia- Caring for Australasians with Renal Impairment (KHA- CARI) and adapting consistently measured method to assess medication nonadherence can be a promising step. Clinicians should be aware of different strategies to promote adherence in this unique patient group, including reducing pill burden, being aware of potential adverse effects of medications which promote nonadherence, and strategies such as using combination products. It is also imperative to improve education regarding patient’s medication regimens, and provide concise instructions to prevent confusion. Future research should be directed towards more rigorous approaches such as prospective longitudinal study design and aim towards developing standard definitions and validating available measurement tools, such as the MRCI, while focusing on the role of additional factors such as psychosocial and behavioural factors in predicting nonadherence to medications.

## Supporting Information

S1 Appendix PRISMA checklist. (DOCX)

S2 Appendix Electronic search strategy. (DOCX)
