# Supplementary material for: Nonadherence to Medication Therapy in Haemodialysis Patients: A Systematic Review
Source: PLoS One. 2015 Dec 4;10(12):e0144119. doi: 10.1371/journal.pone.0144119 (PMC4670103; doi:10.1371/journal.pone.0144119)
Supplement: S2 Appendix — (DOCX) [file pone.0144119.s002.docx]

**S2 Appendix. Electronic search strategy (November, 2014)**

| **ID** | **Query** |
| --- | --- |
| **PubMed Search Strategy** | |
| #1 | ((((((((hemodialysis[MeSH Terms]) OR hemodialysis, home[MeSH Terms]) OR hemodialysis unit, hospital[MeSH Terms]) OR therapy, renal replacement[MeSH Terms]) OR disease, end stage kidney[MeSH Terms]) OR chronic renal failure[MeSH Terms]) OR chronic renal insufficiency[MeSH Terms]) OR chronic kidney failure[MeSH Terms]) OR chronic kidney insufficiency[MeSH Terms] |
|  | AND |
| #2 | ((((adherence, medication[MeSH Terms]) OR adherence, patient[MeSH Terms]) OR compliance, medication[MeSH Terms]) OR compliance, patient[MeSH Terms]) OR concordance |
|  | AND |
| #3 | ((((medication*) OR regimen*) OR schedule*)) OR ((session*) OR exchange*) |
| **Embase Search Strategy** | |
| #1 | (('hemodialysis'/exp/mj OR 'hemodialysis') OR ('end stage renal disease'/exp/mj OR 'end stage renal disease') OR ('chronic kidney failure'/exp/mj OR 'chronic kidney failure') OR ('renal replacement therapy-dependent renal disease'/exp/mj OR 'renal replacement therapy-dependent renal disease')) |
|  | AND |
| #2 | (('drug therapy'/exp OR 'drug therapy') OR ('drug'/exp/mj OR 'drug') OR ('drug dose regimen'/exp OR 'drug dose regimen')) |
|  | AND |
| #3 | (('medication compliance'/exp OR 'medication compliance') OR 'concordance') |
| **CINAHL Search Strategy** | |
| S1 | (MH "Hemodialysis") OR (MM "Renal Replacement Therapy+") OR (MM "Kidney Failure, Chronic+") OR (MM "Renal Insufficiency, Chronic+") |
|  | AND |
| S2 | (MM "Medication Compliance") OR (MM "Patient Compliance+") OR "concordance" |
|  | AND |
| S3 | "medication" OR "drug" OR (MH "Medication Regimen (Omaha)") OR "regimen" OR (MM "Drug Administration Schedule") |
| **PsycInfo Search Strategy** | |
| S1 | hemodialysis OR (hemodialysis unit) OR (renal replacement therapy) OR (end stage kidney disease) OR (end stage kidney failure) OR (chronic renal failure) OR (chronic kidney failure) OR (chronic kidney insufficiency) OR (chronic renal insufficiency) |
|  | AND |
| S2 | adherence OR (medication adherence) OR (patient adherence) OR compliance OR (medication compliance) OR (patient compliance) OR concordance OR (patient concordance) |
|  | AND |
| S3 | medication* OR drug* OR regimen* OR schedule* |
| **Cochrane Search Strategy** | |
| #1 | MeSH descriptor: [Renal Dialysis] this term only |
| #2 | MeSH descriptor: [Hemodialysis Units, Hospital] explode all trees |
| #3 | MeSH descriptor: [Hemodialysis, Home] 1 tree(s) exploded |
| #4 | MeSH descriptor: [Renal Replacement Therapy] this term only |
| #5 | MeSH descriptor: [Kidney Failure, Chronic] this term only |
| #6 | #1 or #2 or #3 or #4 or #5 |
| #7 | MeSH descriptor: [Medication Adherence] explode all trees |
| #8 | MeSH descriptor: [Patient Compliance] this term only |
| #9 | "concordance":ti,ab,kw (Word variations have been searched) |
| #10 | #7 or #8 or #9 |
| #11 | "medication":ti,ab,kw (Word variations have been searched) |
| #12 | "drug":ti,ab,kw (Word variations have been searched) |
| #13 | "regimen":ti,ab,kw (Word variations have been searched) |
| #14 | "schedule":ti,ab,kw (Word variations have been searched) |
| #15 | #11 or #12 or #13 or #14 |
| #16 | #6 and #10 and #15 |
